# Plastid chaperonin proteins Cpn60α and Cpn60β are required for plastid division in *Arabidopsis thaliana*

**DOI:** 10.1186/1471-2229-9-38

**Published:** 2009-04-06

**Authors:** Kenji Suzuki, Hiromitsu Nakanishi, Joyce Bower, David W Yoder, Katherine W Osteryoung, Shin-ya Miyagishima

**Affiliations:** 1 Initiative Research Program, Advanced Science Institute, RIKEN, 2-1 Hirosawa, Wako, Saitama 351-0198, Japan; 2 Department of Plant Biology, Michigan State University, East Lansing, Michigan 48824, USA

## Abstract

**Background:**

Plastids arose from a free-living cyanobacterial endosymbiont and multiply by binary division as do cyanobacteria. Plastid division involves nucleus-encoded homologs of cyanobacterial division proteins such as FtsZ, MinD, MinE, and ARC6. However, homologs of many other cyanobacterial division genes are missing in plant genomes and proteins of host eukaryotic origin, such as a dynamin-related protein, PDV1 and PDV2 are involved in the division process. Recent identification of plastid division proteins has started to elucidate the similarities and differences between plastid division and cyanobacterial cell division. To further identify new proteins that are required for plastid division, we characterized previously and newly isolated plastid division mutants of *Arabidopsis thaliana*.

**Results:**

Leaf cells of two mutants, *br04 *and *arc2*, contain fewer, larger chloroplasts than those of wild type. We found that *ARC2 *and *BR04 *are identical to nuclear genes encoding the plastid chaperonin 60α (ptCpn60α) and chaperonin 60β (ptCpn60β) proteins, respectively. In both mutants, plastid division FtsZ ring formation was partially perturbed though the level of FtsZ2-1 protein in plastids of *ptcpn60β *mutants was similar to that in wild type. Phylogenetic analyses showed that both ptCpn60 proteins are derived from ancestral cyanobacterial proteins. The *A. thaliana *genome encodes two members of ptCpn60α family and four members of ptCpn60β family respectively. We found that a null mutation in *ptCpn60α *abolished greening of plastids and resulted in an albino phenotype while a weaker mutation impairs plastid division and reduced chlorophyll levels. The functions of at least two ptCpn60β proteins are redundant and the appearance of chloroplast division defects is dependent on the number of mutant alleles.

**Conclusion:**

Our results suggest that both ptCpn60α and ptCpn60β are required for the formation of a normal plastid division apparatus, as the prokaryotic counterparts are required for assembly of the cell division apparatus. Since moderate reduction of ptCpn60 levels impaired normal FtsZ ring formation but not import of FtsZ into plastids, it is suggested that the proper levels of ptCpn60 are required for folding of stromal plastid division proteins and/or regulation of FtsZ polymer dynamics.

## Background

All plastids trace their origins to a primary endosymbiotic event in which a previously nonphotosynthetic protist engulfed and enslaved a cyanobacterium. Over time, most of the genes once present in the endosymbiont have been lost or transferred to the host nuclear genome; those nuclear-encoded proteins used by the plastid are translated by the host and targeted back into the organelle to express their functions [1,2]. Consistent with this scenario, plastids are never synthesized *de novo *and they cannot multiply independently. Their continuity is maintained by the division of preexisting plastids, which is performed and controlled by proteins encoded in the nuclear genome [3-6].

Consistent with the endosymbiotic origin of plastids, molecular genetic studies in *A. thaliana *have defined several nucleus-encoded homologs of cyanobacterial cell division proteins that function in plastid division in photosynthetic eukaryotes [7-13]. Plastid division requires assembly of FtsZ1 and FtsZ2, homologs of the tubulin-like bacterial protein FtsZ, into a ring structure at the midplastid division site [14-16]. The FtsZ ring is localized to the midplastid through the activities of MinD and MinE [9-12] and is thought to be stabilized by the J-domain-like protein ARC6 [13]. Mutations in several other cyanobacteria-derived genes, such as *Giant Chloroplast 1 *[17,18] and *Crumpled Leaf *[19], also cause defects in plastid division, although their roles in the division process are still not known.

Plant-specific proteins (dynamin-related GTPase protein, PDV1 and PDV2) also regulate chloroplast division [20-22]. Division involves the assembly and constriction of the endosymbiont-derived FtsZ ring on the stromal surface of the inner envelope membrane and the plant-specific dynamin ring on the cytosolic surface of the outer envelope membrane. This coordination is mediated by the outer envelope spanning proteins PDV1 and PDV2, and inner envelope spanning protein ARC6 [23]. As above, recent studies identified several additional components of the plastid division machinery. However, several other proteins that are involved in bacterial cell division [24] are not found in plants, and there are still unidentified *arc *(accumulation and replication of chloroplasts) loci that impair chloroplast division in *A. thaliana *[25], suggesting that there are still unidentified components of the plastid division machinery. In order to identify new plastid division proteins, we are using forward genetics approaches.

By characterizing plastid division mutants, we found that the cyanobacteria-derived chaperonin proteins ptCpn60α and ptCpn60β are required for proper plastid division in *A. thaliana*. The *A. thaliana *genome encodes several members of the ptCpn60α and ptCpn60β families and our analyses suggest that at least two ptCpn60β proteins have redundant functions. Moderate reduction of ptCpn60β protein levels impaired plastid division while severe loss abolished greening of plastids, suggesting that the level of ptCpn60β is important for proper plastid division. Since chaperonin proteins have been shown to be required for assembly of the division apparatus in bacteria [26,27], their activities in the division machinery are conserved between bacteria and plastids.

## Results

### Mutations in ptCpn60α and ptCpn60β impair plastid division

**Figure 1:**
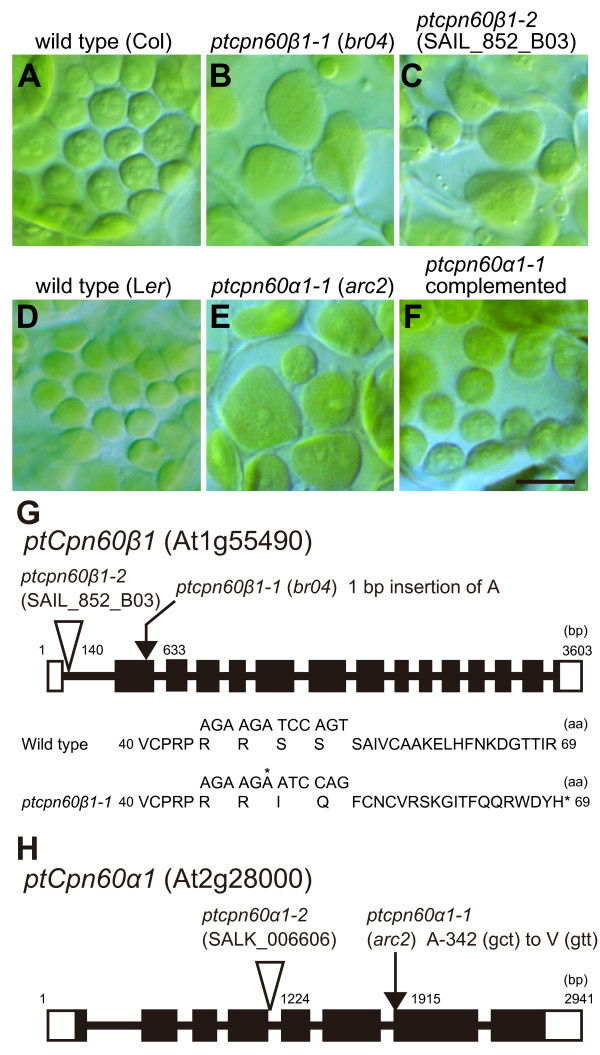
**Chloroplast division defects and mutation sites in plastid *cpn60 *mutants**. (A-F) Chloroplasts in leaf mesophyll cells were observed by Nomarski optics. Since the background of *ptcpn60β1-1 *(*br04*) and *ptcpn60β1–2 *(SAIL_852_B03) is Col-0 and the background of *ptcpn60α1-1 *(*arc2*) is *Ler*, mutants were compared to their respective wild types. Scale bar = 10 μm. (G-H) Schematic diagram of *ptCpn60β1 *and *ptCpn60α1*. Mutation sites of *ptcpn60β1-1 *(*br04*) and *ptcpn60α1-1 *(*arc2*) are indicated by arrows and the positions of T-DNA insertions in *ptcpn60β1–2 *(SAIL_852_B03) and *ptcpn60α1–2 *(SALK_006606) are indicated by triangles. Exons are depicted as black boxes and UTRs are depicted as white boxes. bp, base pair. aa, amino acids.

In order to find new proteins required for plastid division, we screened 22,650 *A. thaliana *Activation Tagging Lines [28]. By microscopic observation of leaf cell chloroplasts, we found twenty-five mutant lines with chloroplasts that were significantly altered in number and size within single cells as compared with those in the wild type [29]. Among these mutants, one line, *br04*, which contained enlarged chloroplasts, was characterized in this study (*ptcpn60β1-1*; Figure 1B). The growth of *br04 *was slightly slower than that of the wild type while the mutant plants were fertile. All the F_1 _progeny, after crossing *br04 *with wild type, displayed normal chloroplast morphology. In F_2 _progeny, the chloroplast-division phenotype segregated in approximately a 3:1 ratio (wild type:*br04*). These results indicated that the chloroplast-division phenotype of *br04 *is recessive and that the phenotype is caused by a mutation in a single genomic locus.

Because the T-DNA insertion in *br04 *did not co-segregate with the mutant phenotype in the F_2 _population, we identified the mutation by map-based cloning. *br04 *bears a single nucleotide insertion in At1g55490, which encodes a plastid chaperonin 60β (ptCpn60β1) (Figure 1G). The nucleotide insertion produced a premature stop codon in the second exon (Figure 1G). To confirm that the mutation in At1g55490 is linked to the chloroplast-division phenotype, we observed another T-DNA insertion mutant in the same gene (SAIL_852_B03; *ptcpn60β1–2*; Figure 1G). The mutant displayed a chloroplast-division defect similar to that of *br04 *(Figure 1C). Because two independent mutant alleles of At1g55490 showed chloroplast division defects, we conclude that At1g55490 is identical to *br04 *and is required for plastid division.

Supporting the above relationship between ptCpn60β and plastid division, map-based cloning of the previously isolated chloroplast division mutant *arc2 *[30] revealed a mutation in At2g28000, which encodes ptCpn60α. Leaf mesophyll cells in *arc2 *mutants contain fewer and larger chloroplasts than those in wild type cells ([30], Figure 1E), similar to *br04*. The *arc2 *mutation bears a single nucleotide substitution, which converts Ala-342 to Val in At2g28000 (Figure 1H). In addition, a genomic copy of At2g28000 complemented the chloroplast-division defect in *arc2 *(Figure 1F), indicating that *ARC2 *is identical to At2g28000. These results indicate that ptCpn60α as well as ptCpn60β proteins are required for normal plastid division.

### Moderate reduction of ptCpn60α or ptCpn60β activity causes defects in chloroplast division while severe reduction abolishes greening

A previous study showed that a T-DNA insertion null mutant of *ptcpn60α *(*schlepperless*, At2g28000) had a defect in embryo development and greening of plastids [31]. In contrast, *arc2*, a missense allele, germinated normally, though it showed a dwarf phenotype later in development. *br04 *(At1g55490) also germinated normally, consistent with previous observations of another null allele of At1g55490, *lesion initiation 1 *(*len1*) [32]. Although leaves of *len1 *had wrinkled irregular surfaces and displayed lesion formation under short-day conditions, under long-day conditions similar to those used throughout our study, these phenotypes were not observed and the plants showed a dwarf phenotype similar to that of *br04 *[32]. We hypothesized that the differences in phenotypes resulted from remnant ptCpn60α activity in the case of *arc2 *and redundant ptCpn60β proteins in the case of *br04*.

**Figure 2:**
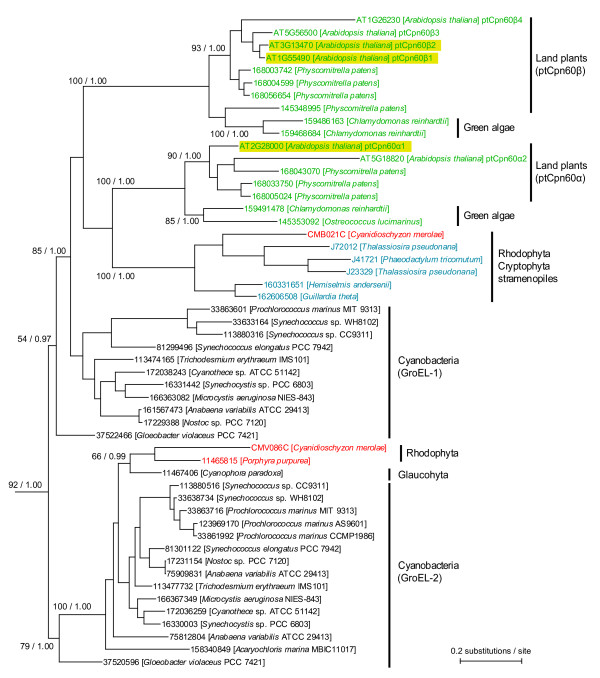
**Phylogenetic relationships among plastid chaperonin 60 proteins**. A phylogenetic tree was constructed using the Maximum-likelihood and Bayesian methods. Sequences from Viridiplantae, Rhodophyta and other eukaryotic groups containing chloroplasts of red algal origin are shown in green, red, and blue, respectively. GI numbers or locus IDs of proteins are shown with names of species. Proteins highlighted by yellow boxes were examined in this study. Bootstrap values by RaxML [57] and posterior probability values by MrBayes [56] are indicated at the branch nodes. Only the clades containing cyanobacterial and plastid proteins are shown; the whole tree is shown in Additional file 1.

To address the above possibilities, we first analyzed ptCpn60 proteins in *A. thaliana *by phylogenetic analyses. The results showed monophyly of six *A. thaliana *proteins with cyanobacterial chaperonin 60 proteins (Figure 2). Of these, two proteins, including At2g28000, were grouped as ptCpn60α (we named them ptCpn60α1 and ptCpn60α2), and four proteins, including At1g55490, were grouped as ptCpn60β (ptCpn60β1 through ptCpn60β4). These results are consistent with a previous classification [33]. Further, our phylogenetic analyses indicated that there are two types of cyanobacterial chaperonin 60 proteins, GroEL-1 and GroEL-2, and that only one group, GroEL-1, gave rise to ptCpn60α and ptCpn60β in land plants and green algae (Figure 2).

**Figure 3:**
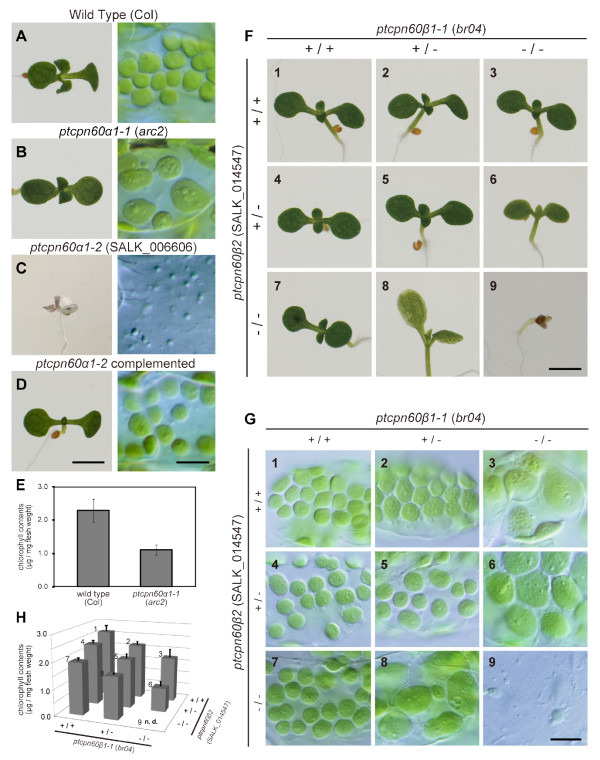
**Comparison of phenotypes between two *ptcpn60α1 *mutants and in combinations with *ptcpn60β1-1 *and *ptcpn60β2 *mutants**. (A-E) Seedlings, chloroplasts in leaf mesophyll cells, and chlorophyll contents of *ptcpn60a1 *mutants. Phenotypes of *ptcpn60α1–2 *(SALK_006606) were complemented by a *ptCpn60α *transgene (D). (F-H) The seedlings, chloroplasts in leaf mesophyll cells, and chlorophyll contents in plants with combinations of *ptcpn60β1-1 *and *ptcpn60β2 *mutations. +/+, wild type. +/-, heterozygous mutant. -/-, homozygous mutant. Scale bars = 2 mm (A-D, left panels), 10 μm (A-D, right panels), 2 mm (F), and 10 μm (G). Error bars represent the standard deviation (E, H). n.d., not determined (H).

Next, we compared the phenotypes of *arc2 *(a missense allele, *ptcpn60α1-1*) and a T-DNA insertion mutant (*ptcpn60α1–2*, SALK_006606, Figure 1H) of *ptCpn60α1 *(At2g28000) (Figures 3A to 3C). In contrast to *arc2*, seedlings of the insertion mutant exhibit an albino phenotype and the growth of this mutant was severely suppressed (Figure 3C), similar to that of *schlepperless*, another T-DNA insertion null allele [31]. By microscopy, we observed small and colourless plastid-like organelles in leaf cells, but no developed chloroplasts (Figure 3C). When a genomic fragment bearing the *ptCpn60α1 *(At2g28000) gene was introduced into the mutant, the phenotype was complemented (Figure 3D). These results indicate that loss of ptCpn60α1 abolishes greening of plastids. In support of this conclusion, the amount of chlorophyll extracted from true leaves of *arc2 *was less than that of wild type (Figure 3E), although the *arc2 *cells contained green chloroplasts that showed defects in division (Figure 3B). The above observations of two *ptcpn60α1 *mutants suggest that complete loss of ptCpn60α1 activity fully abolishes greening of plastids while the weaker *arc2 *allele, though probably retaining residual activity of ptCpn60α1, still confers chloroplast division defects.

We addressed possible functional redundancy among ptCpn60β proteins. The phylogenetic analyses showed that ptCpn60β1 (*br04*, At1g55490) has the closest evolutionary relationship with ptCpn60β2 (At3g13470) (Figure 2) and a BLAST search showed 92% identity between the two amino acid sequences. In order to assess whether ptCpn60β2 protein is also required for plastid division and/or plastid development and whether the functions of ptCpn60β1 and ptCpn60β2 proteins are redundant, we observed a T-DNA insertion mutant of ptCpn60β2 (SALK_014547, *ptcpn60β2*). Although the mutant did not exhibit plastid division or embryo development defects (Figures 3F7 and 3G7), the *ptcpn60β1-1 *(*br04*) *ptcpn60β2 *(SALK_014547) double mutant exhibited small, albino seedlings (Figures 3F9 and 3G9), similar to the *ptcpn60α1 *T-DNA mutant (*ptcpn60α1–2*, Figure 3C). Since the *ptcpn60β1-1 *(*br04*) and *ptcpn60β2 *(SALK_014547) single mutants did not show the albino phenotype (Figures 3F3 and 3F7), the above results indicate that ptCpn60β1 and ptCpn60β2 are redundant.

To further examine the redundancy between the two ptCpn60β proteins with regard to plastid division and greening, we observed all possible combinations of the *ptcpn60β1-1 *and *ptcpn60β2 *mutations (i.e. combinations of wild-type, heterozygous and homozygous mutations) (Figures 3F to 3H). All combinations except the double homozygote germinated normally (Figure 3F), while leaf chlorophyll content was reduced depending on the number of mutant alleles (Figure 3H).

Similar to the chlorophyll content, the plastid division defect was dependent on the number of mutant alleles (Figure 3G). Other than the double homozygous mutant, all combinations containing the *ptcpn60β1-1 *homozygous mutation (Figures 3G3 and 3G6) and the combination of the *ptcpn60β1-1 *heterozygous mutation and *ptcpn60β2 *homozygous mutation (Figure 3G8) showed a large-chloroplast phenotype, while the size and number of chloroplasts were normal in other combinations (Figures 3G1, G2, G4, G5 and 3G7). These results suggest that ptCpn60β1 and ptCpn60β2 have redundant functions in plastid division. Similarly to *ptcpn60α *(Figures 3B and 3C), severe mutation of ptCpn60β fully abolishes greening of plastids. In contrast, weaker mutations in ptCpn60β partially affect greening while chloroplast division is defective even under these conditions.

### ptCpn60α and ptCpn60β are required for proper FtsZ ring formation

**Figure 4:**
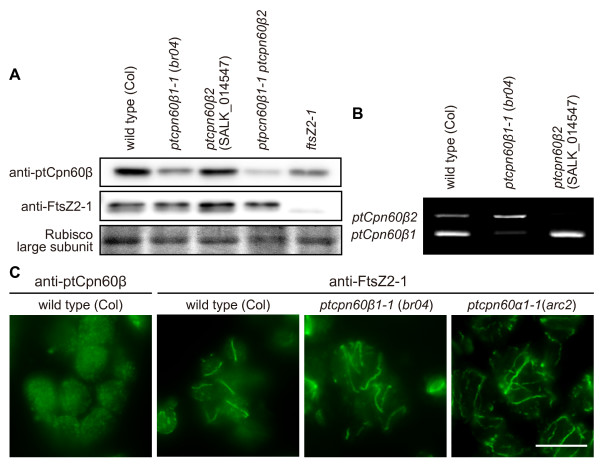
**Expression and localization of ptCpn60β and FtsZ in plastid *cpn60 *mutants**. (A) Immunoblot analyses using anti-ptCpn60β and anti-FtsZ2-1 antibodies. Total proteins extracted from seedlings of wild type, *ptcpn60β1-1*, *ptcpn60β2*, *ptcpn60β1-1 ptcpn60β2*, and *ftsZ2-1 *in mesophyll cells were blotted. (B) RT-PCR analyses comparing transcript levels of *ptCpn60β1 *and *ptCpn60β2*. cDNA was prepared from total RNA extracted from the wild type, *ptcpn60β1-1*, and *ptcpn60β2*. (C) Localization of ptCpn60β in wild type and of FtsZ2-1 in wild type, *ptcpn60β1-1*, and *ptcpn60α1-1 *in mesophyll cells was examined by immunofluorescence microscopy. Scale bars = 10 μm.

To confirm the reduction of ptCpn60β proteins in *ptcpn60β *mutants and further examine localization of the proteins, we prepared antibodies using recombinant ptCpn60β1. On immunoblots, the antibodies detected a single band of ~60 kDa, close to the predicted size of the ptCpn60β proteins (the predicted transit peptide was omitted for calculation of the molecular mass, Figure 4A). When the same amount of total protein extracted from whole plants was examined, the intensity of the band was reduced in the *ptcpn60β1-1 *mutant (*br04*) relative to that in the wild type. However, a residual band was detected, similar to a previous report that anti-spinach ptCpn60β antibodies recognized residual ptCpn60β in the *ptcpn60β1-1 *mutant (*len1*, null mutant) [32]. The intensity of the residual band was further reduced in the *ptcpn60β1-1 ptcpn60β2 *double mutant (Figure 4A), suggesting that the antibodies recognize both ptCpn60β1 and ptCpn60β2 (predicted sizes of ptCpn60β1, ptCpn60β2, ptCpn60β3, and ptCpn60β4 without their transit peptides are 547, 547, 567, and 574 amino acids, respectively [33].) and confirming reduction of total ptCpn60β protein level in these mutants. Since the antibodies still detected a faint band at the same position on the gel in the double mutant (Figure 4A), they may also recognize ptCpn60β3 and/or ptCpn60β4. In the immunoblot analysis, there was little difference in the intensity of the band between wild type and *ptcpn60β2*. This is probably because the ptCpn60β2 protein level is lower than the levels of the other ptCpn60β proteins, as suggested by RT-PCR analyses showing that the *ptCpn60β2 *transcript level is lower than that of *ptCpn60β1 *(Figure 4B).

In contrast to the reduction of ptCpn60β, levels of ribulose-1,5-bisphosphate carboxylase/oxygenase (Rubisco) large subunit and FtsZ2-1 were not altered in the *ptcpn60β *mutants (Figure 4A). In addition, the size of FtsZ2-1 in the mutant was the same as that of mature protein in the wild type. These results indicate that nucleus-encoded FtsZ2-1 is properly imported into the plastids and processed in the *ptcpn60β *mutants.

In order to examine the relationship between ptCpn60 proteins and chloroplast division, we first examined the localization of ptCpn60β by immunofluorescence microscopy using the anti-ptCpn60β antibodies in the wild type. The fluorescence signal was detected specifically in chloroplasts but was not detected by preimmune antisera or secondary antibodies alone (not shown). The fluorescence signal detected by the antibodies was spattered throughout the chloroplasts and no specific localization at the division site was observed (Figure 4C).

To assess how *ptcpn60 *mutations affect the chloroplast division machinery, we examined the localization of the chloroplast division FtsZ proteins in the *ptcpn60α1-1 *and *ptcpn60β1-1 *mutants by immunofluorescence microscopy using anti-AtFtsZ2-1 antibodies. In the wild type, FtsZ2-1 localizes in a single ring at the chloroplast division site as reported previously (Figure 4B, [14]). In contrast, the enlarged chloroplasts in both the *ptcpn60β1-1 *(*br04*) and *ptcpn60α1-1 *(*arc2*) mutants contained abnormally long, disordered FtsZ filaments (Figure 4C), indicating that FtsZ ring formation is perturbed in both mutants.

## Discussion

In this study, we showed that both ptCpn60α and ptCpn60β are required for plastid division as well as for greening of plastids. The results also indicate that the mutant phenotypes vary depending on the severity of the mutations. In addition to defects in plastid division, *ptCpn60 *mutants exhibited dwarfed or other developmental defects [31,32] (Figure 3). A similar situation was observed in the *crumpled leaf *(*crl*) mutant, which also showed both plastid division defects and abnormal development [19]. Because both ptCpn60 and CRL [19] function in chloroplasts and, in our screening, several mutant plants that showed abnormal morphology contained chloroplasts of normal size, it is unlikely that the developmental defects are the cause of the observed chloroplast division defects.

Chaperonins are evolutionarily conserved molecular chaperones found in bacteria (named GroE), mitochondria and plastids. The structure and mechanisms of chaperonin function have been well studied mainly using the chaperonin of *Escherichia coli*, GroE [34]. The GroE chaperonin functions as a large complex consisting of multiple 60-kD GroEL and 10-kD GroES subunits [34]. Although *in vitro *studies have clarified the mechanism of GroEL as a molecular chaperone, the *in vivo *roles are poorly understood. GroE is essential for the viability of *E. coli *[35] and this is partly because GroE is required for cell wall synthesis. In addition to the cell lysis phenotype of GroE-depleted *E. coli*, it has been reported that cells with impaired GroE exhibit filamentous cell morphology owing to defects in cell division [27]. The filamentous phenotypes were also observed in GroE-depleted *Caulobacter crescentus *and *Streptococcus mutans*, suggesting that GroE plays a universal role in cell division in bacteria [36,37].

Plastid Cpn60 proteins are homologs of bacterial GroEL and phylogenetic studies indicate that plant Cpn60 proteins evolved from GroEL proteins in the cyanobacterial ancestor of plastids ([33], Figure 2). Previous studies showed that depletion of ptCpn60 proteins in *A. thaliana *results in abnormal development of embryos and plastids [31] and cell death in some growth conditions [32]. Although severe mutations in *ptCpn60 *genes resulted in albino and dwarf seedlings, we found that weaker mutations confer defects in plastid division. Even though plastid chaperonins are expected to be involved in several processes occurring in plastids as are bacterial chaperonins, our results suggest that one of the roles of the chaperonins is related to plastid division and that the role in division is conserved between bacteria and plastids. It is also known that plastid chaperonins are different from *E. coli *GroE in that plastids contain two distinct proteins, ptCpn60α and ptCpn60β, both of which are expressed in all tissues [31,38]. Despite the difference, our results showed that both ptCpn60α and ptCpn60β are required for plastid division.

In our analyses, depletion of ptCpn60 proteins did not alter the level of plastid FtsZ. The size of the FtsZ protein in the mutants was the same as that in wild type, indicating that the transit peptide was cleaved and the protein was imported into plastids. Both in *E. coli *[26] and *A. thaliana *chloroplasts (Figure 4B), normal FtsZ ring formation is impaired in the respective chaperonin mutants even though FtsZ protein levels are normal (Figure 4A). These results suggest that ptCpn60 proteins are not required for import of plastid division proteins into plastids. Rather, it is suggested that ptCpn60s are required for assembly and/or maintenance of the plastid division apparatus after import of the components into plastids [8-10,13]. However, the mechanistic basis of the chloroplast division defect remains unclear. The abnormally long, disorganized FtsZ filaments observed in the *ptcpn60 *mutants resemble the reported FtsZ2 localization patterns in an *ftsZ1 *null mutant [39], an *ftsZ1 *antisense line [14], and in a line overexpressing *ARC6*, which functions in part to stabilize FtsZ polymers [13]. The *ptcpn60 *FtsZ morphologies are distinct from those observed in *minD *[40] (multiple closed FtsZ rings with multiple constriction), *minE *[40], *arc6 *[13] (many fragmented short FtsZ filaments), *pdv1, pdv2 *and *arc5 *[22] (multiple rings or spirals at the constriction site) mutants. The mutant phenotypes suggest that reduced ptCpn60 levels result in excessively stable FtsZ filaments, though whether this is through a direct effect on FtsZ or a regulator of FtsZ assembly, and whether the effects result from misfolding of some proteins in the mutant backgrounds or loss of another activity of ptCpn60, remain to be determined. Whatever the mechanism, the results provided evidence of a role for the ptCpn60 chaperone system in the regulation of FtsZ polymer dynamics *in vivo*.

We compared the effects of several combinations of *ptCpn60β *alleles. The appearance of the chloroplast division phenotype depends on the number of disrupted alleles of *ptcpn60β*. For example, chloroplast size and number in the *ptcpn60β1-1 *heterozygote and *ptcpn60β2 *homozygote were normal, but combining these alleles (*ptcpn60β1-1 *heterozygous *ptcpn60β2 *homozygous mutant) impaired chloroplast division (Figure 3G8). The lack of an obvious phenotype in *ptcpn60β2 *is probably because the level of total ptCpn60β decreased little in this mutant (Figure 4C). The results suggest that ptCpn60β1 and ptCpn60β2 have redundant functions and that the plastid division defects in the *ptcpn60β *mutants are due to decreased ptCpn60β dosage. Thus far, several plastid division proteins of cyanobacterial origin, such as FtsZ, MinD, MinE, ARC6, and GC1, were identified [7-13,17]. Studies showing that the stoichiometry among these proteins is tightly maintained in plants [41] and that moderate loss or overexpression of FtsZ, MinD and MinE impairs plastid division [11,42,43] suggest that normal plastid division requires the presence of the proper stoichiometric relationship among plastid division proteins. The observed defects in plastid division in a series of *ptcpn60β *mutants even in the presence of wild type *ptCpn60β *alleles (Figure 3G) may reflect disruption of the stoichiometric relationship of functional plastid division proteins due to misfolding in the mutants after import from the cytosol.

Studies using *E. coli *showed preferential localization of a population of GroEL at division sites by immunofluorescence labelling [26]. In our immunofluorescence analyses, however, ptCpn60β proteins are spattered throughout the chloroplasts of *A. thaliana *and we could not observe predominant localization of the protein at the division site. This observation is perhaps because of the existence of several chloroplast proteins which require Cpn60 proteins for their folding. In fact, many proteins other than division proteins have been identified as possible targets of bacterial GroEL as below. Despite of this observation, it is still possible that a portion of the ptCpn60 pool interacts with the plastid division machinery. A study in *E. coli *further suggested that the division protein FtsE is a target substrate of the GroE system [27]. In contrast, FtsE is missing in plant and algal genomes [44], suggesting that the plastid Cpn60 system targets a different plastid division substrate(s). Proteome-based analyses in *E. coli *identified ~300 proteins that interact with GroE, including the cell division proteins FtsE, FtsA, FtsI, and FtsZ [45-47] although GroE-dependent folding of FtsA, FtsI, and FtsZ has not been examined. Of these, only FtsZ is conserved in plant genomes, raising the possibility that FtsZ might be a target of Cpn60 in the plastid.

Several other molecular chaperone proteins have been shown to function in plastids, such as HSP100 [48] and HSP70 [49], but there is limited information about their substrates [50]. Although it is known that functional specificity of Hsp70 is mediated by specialized co-chaperones, how and what kinds of proteins GroE/Cpn60 recognize *in vivo *is little understood [51]. Further studies on the interaction between GroEL and plastid division proteins *in vivo*, such as co-immunoprecipitation and FRET analyses, would shed light on the role of ptCpn60 in the assembly and/or maintenance of the plastid division machinery.

## Conclusion

Our results show that cyanobacteria-derived ptCpn60α and ptCpn60β proteins are required for plastid division. FtsZ ring formation in plastids, but not import of FtsZ into the plastids, was perturbed in *ptcpn60a *and *ptcpn60β *mutants, suggesting that ptCpn60 proteins are required for assembly of the cyanobacteria-derived part of the plastid division machinery subsequent to import of plastid division proteins, all of which are encoded in the nucleus. Although plants have several members of the ptCpn60α and ptCpn60β family, we found that moderate reduction of ptCpn60 level results in impaired plastid division and reduction of chlorophylls. The results suggest the existence of mechanisms that regulate the levels of the ptCpn60 family of proteins in plastids.

## Methods

### Plant Materials and Growth Conditions

The T-DNA insertion lines SAIL_852_B03 and SALK_014547 were provided by the Arabidopsis Biological Resource Center (ABRC). Seeds were surface-sterilized, sown on Murashige and Skoog agar plates, and stratified at 4°C for 48 h in the dark before germination. All plants were grown in growth chambers under white fluorescent light (a cycle of 16-h light/8-h dark) at 21°C. Seedlings were transferred to soil 2 to 4 weeks after germination and were grown under the same conditions.

### Isolation of *br04 *Mutant

*A. thaliana *Activation Tagging Lines ([28]; provided by RIKEN BioResource Center) were germinated and grown for 3 weeks as described above. Tips of expanding leaves were cut and chloroplasts were observed with Nomarski differential interference optics. Among 22,650 lines observed, the size and number of chloroplasts were significantly altered in 25 lines compared to those in the wild type [29]. One recessive mutant was analyzed further in this study.

### Map-Based Cloning of *br04 *and *arc2*

The *br04 *and *arc2 *[30] mutations were mapped with molecular markers based on a cleaved amplified polymorphic sequence [52] and simple sequence length polymorphisms [53]. We used some markers listed on The Arabidopsis Information Resource (TAIR; ); other markers were designed based on polymorphisms listed at TAIR  in the Monsanto SNP and *Ler *Sequence Collection.

The *BR04 *(Col-0 background) homozygous mutant was crossed with Landsberg *erecta *wild-type plants to generate a mapping population. Analyses using 24 F_2 _progeny with the *br04 *phenotype showed that the mutation is located in a region of 1.26 Mb on chromosome 1 (between polymorphisms CER 458759 and CER 460336). Using 600 F_2 _plants, we fine-mapped the *br04 *mutation to a 112 kb region on chromosome 1, which contains 28 genes (between polymorphisms CER 479886 and CER 446782). The *br04 *mutation was found in At1g55490 by sequencing.

*arc2 *(*Ler *background) was crossed with Col-0 wild-type plants to generate a mapping population of 308 F_2 _mutants identified based on their pale phenotype and enlarged chloroplasts. The pale phenotype was confirmed by measuring relative chlorophyll levels *in planta *using a Minolta SPAD-502 chlorophyll meter [54]. We mapped the *arc2 *locus to a 129 kb region on chromosome 2, which contains 18 genes. The *arc2 *mutation was found in At2g28000 by sequencing.

### DNA Constructs and Plant Transformation

For the *arc2 *complementation construct, a genomic fragment containing the annotated *ARC2 *open reading frame flanked by 1.2 kb at the 5' end and 0.4 kb at the 3' end was amplified by PCR using the primers 5'-CGTTTCAATCACAACCACTCA-3' and 5'-AGTGGTTCCAACGAGTCTGA-3'. A gel-purified fragment was cloned into pGEM-T Easy vector (Promega), excised with *Not*I, and then transferred into pMLBART [14]. The final construct was transformed into *arc2 *plants.

All constructs were transferred to *Agrobacterium tumefaciens *and introduced into *A. thaliana *plants as described [22]. T_1 _plants were selected by resistance to glufosinate and used for further analyses.

### Microscopy

For observation of chloroplast size, tips from expanding leaves were cut and fixed with 3.5% glutaraldehyde in water for 1 h at room temperature and then incubated in 0.1 M Na2-EDTA pH 9.0, for 30 min at 55°C. Samples were analyzed with Nomarski differential interference contrast optics.

Localization of ptCpn60β and FtsZ2-1 was examined by immunofluorescence microscopy using anti-ptCpn60β and anti-FtsZ2-1 antibodies as described [14].

### Phylogenetic analyses

Deduced amino acid sequences encoded by the 82 *GroEL *and Cpn60 *genes *(gi numbers or locus IDs are indicated in Figure 2 and Additional file 1) were aligned using CLUSTAL W [55] and the alignment was refined manually. Gaps were deleted and 490 conserved sites were used for the phylogenetic analyses. Bayesian inference was performed with the program MrBayes version 3.1.2 [56] with WAG+I+G4 model. For the MrBayes consensus trees, 1,000,000 generations were completed with trees sampled each 1,000 cycles. Maximum likelihood trees were constructed using RaxML version 7.0.4 [57] with the WAG matrix of amino acid replacements assuming a proportion of invariant positions and four gamma-distributed rates (WAG+I+G4 model). The local bootstrap probability of each branch was calculated by 100 replications.

### Measurement of chlorophyll content

Chlorophyll was extracted from true leaves of ~5 week-old plants in chilled 80% acetone. Chlorophyll content was measured spectrophotometrically as described [58].

### Analyses of gene expression by RT-PCR

Total RNA of *A. thaliana *was extracted from ~3 week-old plants using an RNeasy Mini Kit (Qiagen). DNase-treated RNA was reverse-transcribed with oligo dT (15) primer, and the resulting cDNA was used as template for PCR. The same regions (the same size) of the *ptCpn60β1 *and *ptCpn60β2 *cDNAs were simultaneously amplified by the same primer set (5'-AAGCTCTCTGGTGGAGTTGC-3' and 5'-CCTGAGTTGTCCATTGGGTT-3'). In order to distinguish the two products, amplified cDNA was treated with *Cla*I, which cuts *ptCpn60β1 *but not *ptCpn60β2 *because of a polymorphism between the two sequences.

### Antibodies and Immunoblot analyses

Anti-ptCpn60β polyclonal antibodies were raised in rabbits using recombinant proteins. A fragment encoding amino acids 45–600 was amplified from cDNA using the primers 5'-CACCGCAGCAAAGGAATTACATTTCA-3'and 5'-CCGTTTCAATATTAGCCTATCTCCTC-3'. A gel-purified fragment was cloned into the TOPO cloning vector (Invitrogen) and 6 × His fusion polypeptides were expressed in *Escherichia coli*, purified and used as antigens. Anti-ptCpn60β was affinity-purified from antisera using the recombinant ptCpn60β coupled to a HisTrap NHS-activated HP (GE Healthcare).

SDS-PAGE and immunoblotting were carried out as described previously [22].

## Authors' contributions

SM and KWO designed the study. KS, HN, and SM screened *A. thaliana *tagging lines, isolated *br04*, mapped the mutation and analyzed the mutant. JB and DWY mapped the *arc2 *locus and analyzed the mutant. KS, DWY, KWO, and SM wrote the manuscript. All authors read and approved the final manuscript.

## Supplementary Material

Additional File 1 **Phylogenetic relationships among chaperonin 60 proteins**. Proteins not shown in Figure 2 (mitochondrial chaperonins and proteins of bacteria other than cyanobacteria) are shown here. Click here for file
