# Supplementary figures and images for: Plastid chaperonin proteins Cpn60α and Cpn60β are required for plastid division in Arabidopsis thaliana
Source: BMC Plant Biol. 2009 Apr 6;9:38. doi: 10.1186/1471-2229-9-38 (PMC2670834; doi:10.1186/1471-2229-9-38)

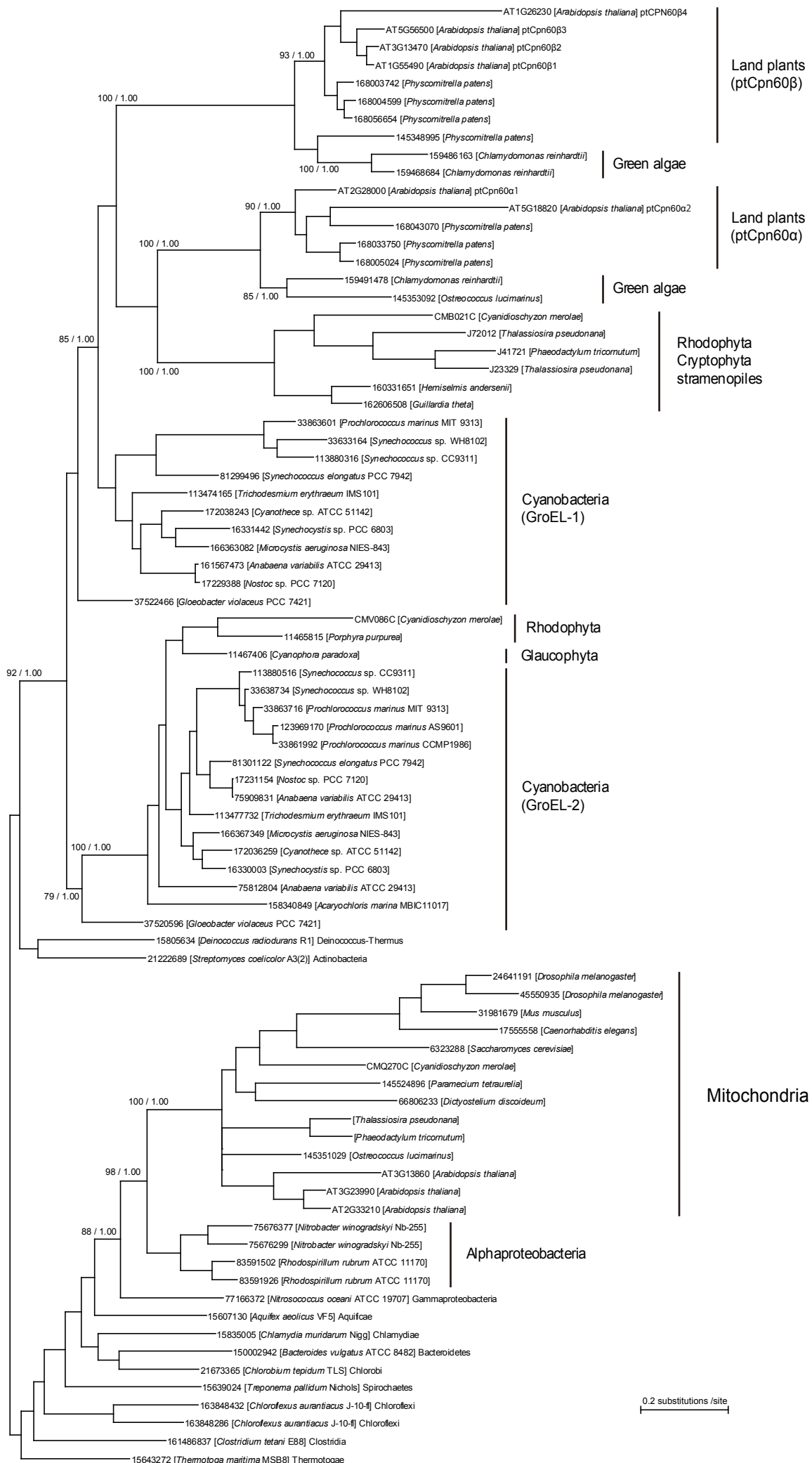

Supplement: Additional File 1 — Phylogenetic relationships among chaperonin 60 proteins. Proteins not shown in Figure 2 (mitochondrial chaperonins and proteins of bacteria other than cyanobacteria) are shown here. [file 1471-2229-9-38-S1.pdf]
